# Malignant Proliferating Trichilemmal Tumor of the Scalp Treated With Excision and Adjuvant Radiotherapy

**DOI:** 10.7759/cureus.116318

**Published:** 2026-09-15

**Authors:** Othmane Bouanani, Mohamed Ait Erraisse, Assia Halmoune

**Affiliations:** 1 Faculty of Medicine, Euromed University of Fez, Fez, MAR; 2 Department of Radiotherapy, Centre Hospitalier Universitaire Hassan II, Fez, MAR

**Keywords:** case based learning, malignant cyst, malignant proliferating trichilemmal tumor, scalp tumor, surgical case access, trichilemmal cyst

## Abstract

Proliferating trichilemmal cysts are common benign lesions, but their malignant counterpart, malignant proliferating trichilemmal tumor (MPTT), is an extremely rare adnexal malignancy that predominantly arises on the scalp. Due to clinical and histological overlap, it is frequently misdiagnosed as squamous cell carcinoma. We report the case of a 70-year-old woman who presented with a progressively enlarging, painless, and bleeding midline scalp mass evolving over one year. A craniofacial CT scan (SOMATOM, Siemens Healthineers, Erlangen, Germany) revealed a well-defined, heterogeneous soft-tissue lesion measuring 75 × 55 mm without underlying bone lysis. Systemic staging showed no distant metastases. An initial incisional biopsy suggested squamous cell carcinoma. The patient underwent wide surgical excision with 1-cm safety margins and deep periosteal bed re-excision. Tension-free primary closure was achieved following extensive scalp undermining and galeal scoring. Definitive histopathological and immunohistochemical examination confirmed MPTT with clear margins. Due to the tumor size and high mitotic rate, the patient received adjuvant hypofractionated Volumetric Modulated Arc Therapy (VMAT; Elekta AB, Stockholm, Sweden), delivering 45 Gy in 10 fractions, resulting in a favorable clinical outcome without recurrence at six months of follow-up. While short-term results demonstrate a favorable outcome, extended surveillance is mandatory due to the potential for late recurrence or metastasis. This case highlights the critical importance of accurate clinical, radiological, and especially histopathological diagnosis using immunohistochemistry to differentiate MPTT from squamous cell carcinoma, ensuring optimal surgical margins and appropriate adjuvant management.

## Introduction

Trichilemmal cysts were first described by Wilson Jones in 1966 [1], while the malignant form of the lesion, officially designated as MPTT, was initially reported by Saida et al. in 1983 [2]. These rare cutaneous adnexal tumors usually present as a long-standing, slow-growing, and painless mass arising on the scalp, typically occurring in female patients between 70 and 80 years of age [3]. The primary clinical and histopathological differential diagnosis is squamous cell carcinoma (SCC); however, distinguishing between these two distinct entities is essential, as MPTT exhibits locally aggressive behavior and carries a well-documented risk for locoregional recurrence as well as distant metastases [4].

The cornerstone treatment of MPTT is primarily surgical, requiring complete wide excision of the mass with adequate safety margins to achieve clear boundaries, while adjuvant therapeutic options such as radiotherapy and chemotherapy may also be indicated in selected high-risk cases [3]. In this report, we present the case of a female patient in her seventh decade of life who underwent wide surgical excision of a large MPTT of the scalp, followed by postoperative hypofractionated radiotherapy, achieving a favorable therapeutic outcome. This case highlights the critical importance of accurate clinical, radiological, and especially histopathological diagnosis to ensure optimal multidisciplinary management and prevent disease complications and recurrence.

## Case presentation

We report the case of a 70-year-old female patient with no significant medical history, who presented with a one-year history of a progressively enlarging, painless, and bleeding midline scalp mass. Initial clinical examination revealed a reddish, pedunculated, exophytic mass with a soft, painless consistency, mobile relative to the deep plane, measuring approximately 7 cm in its greatest dimension, with no cervico-parotid lymphadenopathy (Figure 1).

**Figure 1:**
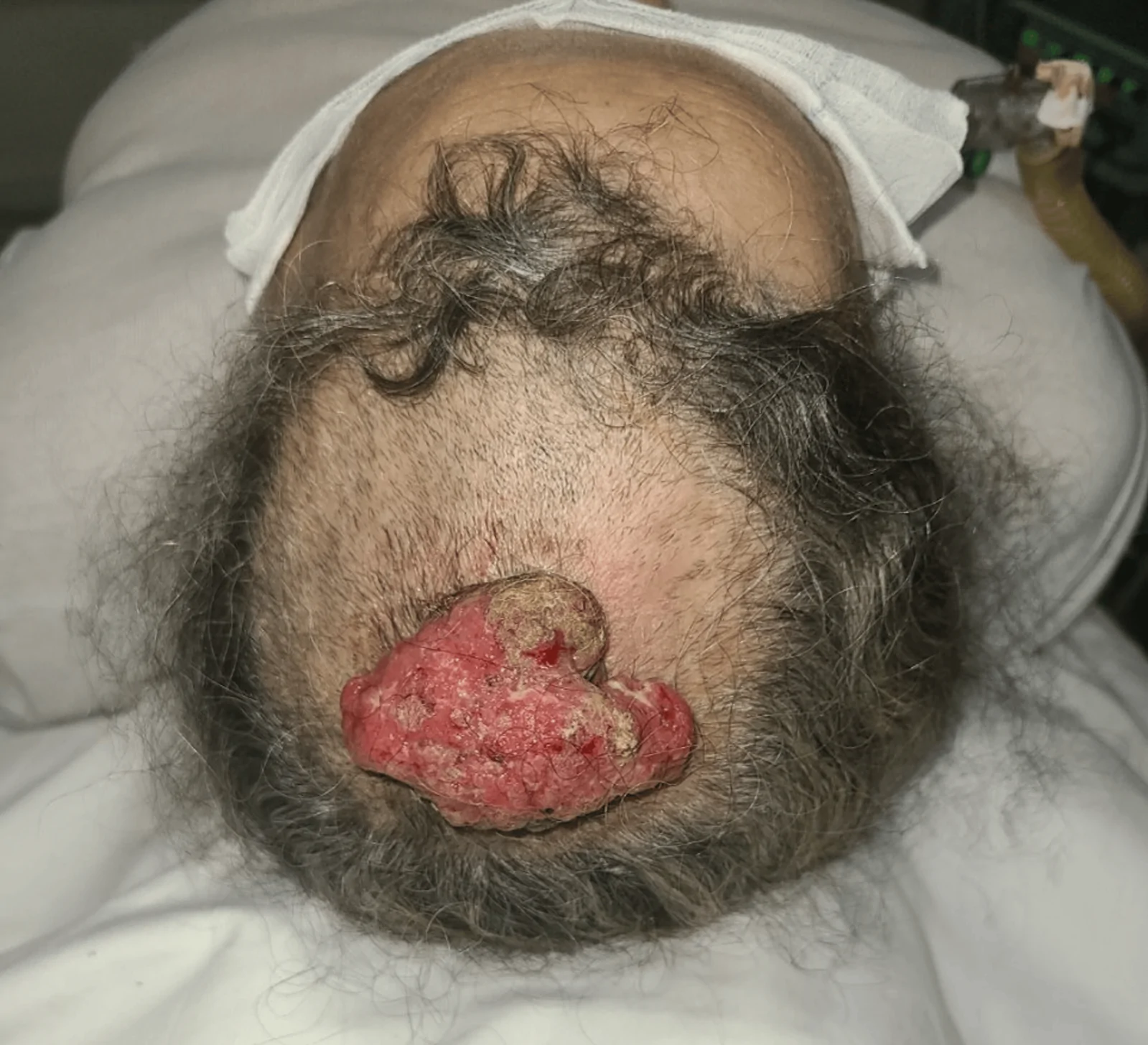
Exophytic scalp mass measuring approximately 70 mm in its greatest dimension.

A craniofacial CT scan (SOMATOM, Siemens Healthineers, Erlangen, Germany) was performed and showed a well-defined, spontaneously hyperdense and heterogeneous scalp lesion measuring 75 × 55 mm, displaying heterogeneous contrast enhancement, with no underlying bone lysis (Figure 2). Systemic extension workup (chest, abdominal, and pelvic CT) showed no evidence of distant metastasis or regional lymphadenopathy.

**Figure 2:**
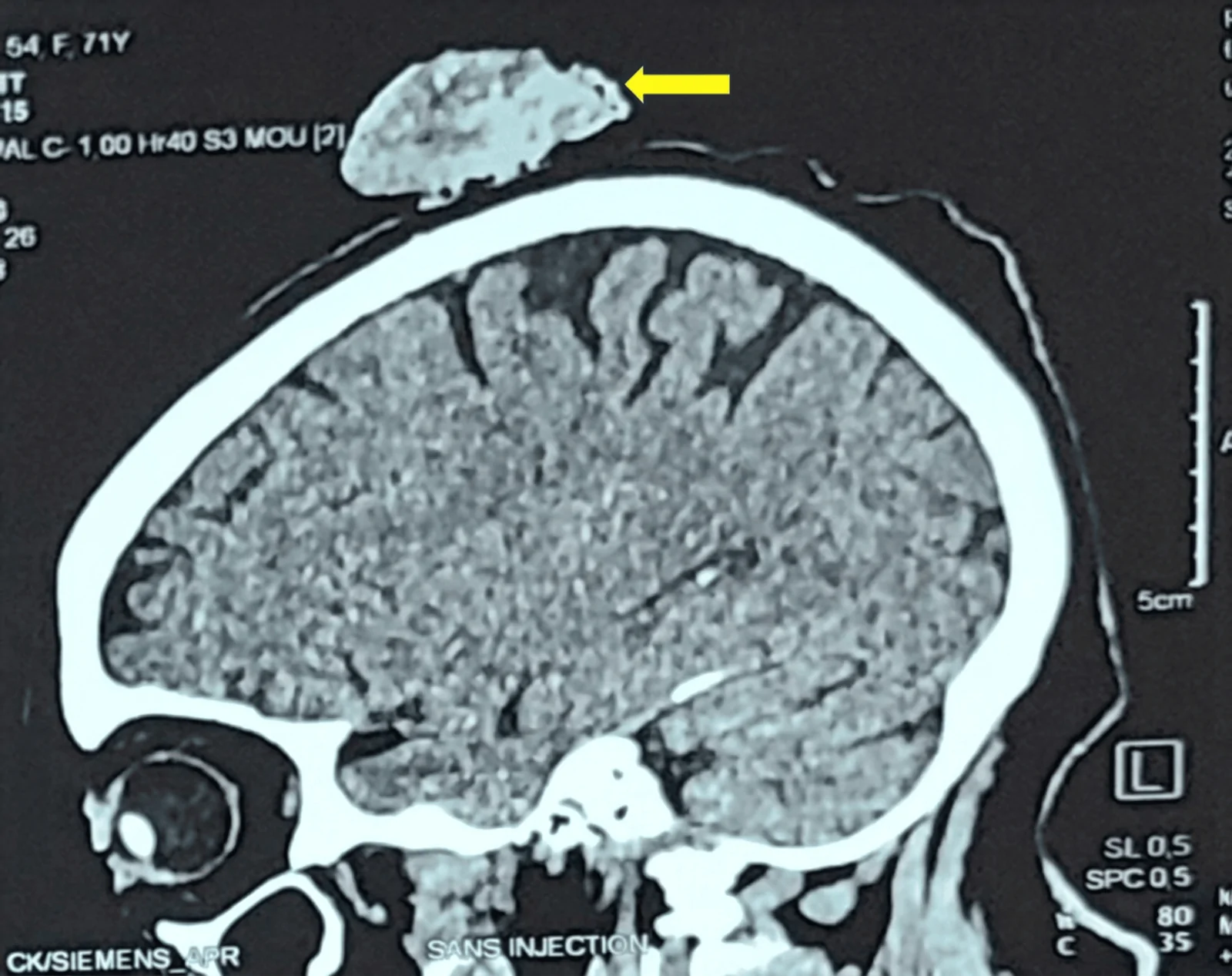
Sagittal CT scan showing a heterogeneous scalp mass measuring 75 × 55 mm (yellow arrow), without underlying bone osteolysis.

An initial incisional biopsy was performed, which was in favor of SCC. The patient subsequently underwent wide surgical excision with a 1-cm safety margin, during which lateral margins and deep periosteal margins were also re-excised to guarantee clear margins. Defect reconstruction was achieved via primary closure. To ensure a tension-free adaptation of the wound edges, wide undermining of the scalp in the avascular subgaleal plane and multiple parallel incisions (scoring) of the galea were performed. Hemostasis was carefully verified intraoperatively, and a suction drain was placed (Figure 3).

**Figure 3:**
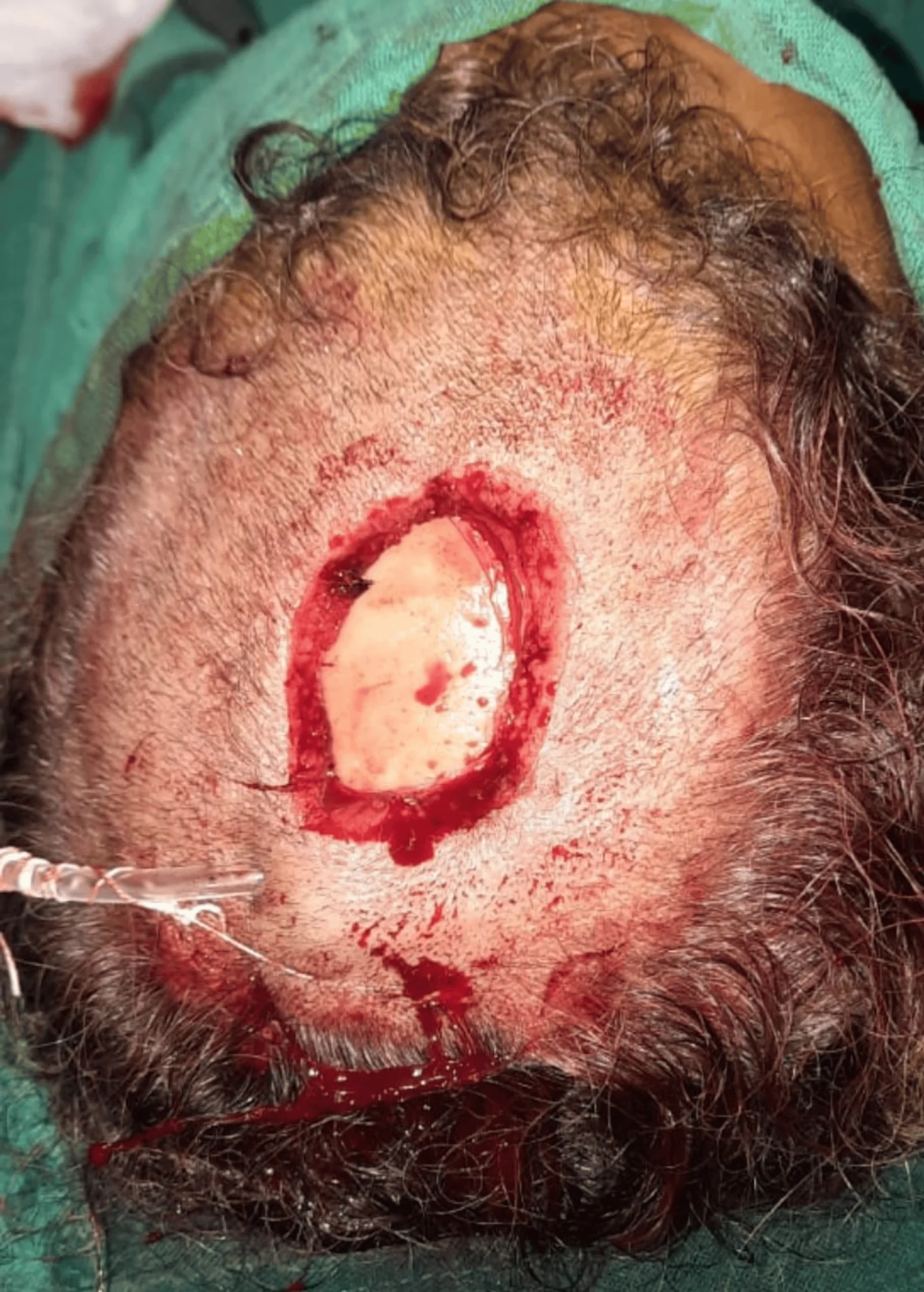
Intraoperative view showing the scalp defect following tumor resection

Postoperative recovery was uneventful, and the patient was discharged the following day. Definitive histopathological examination of the surgical specimen confirmed MPTT with complete excision and clear lateral and deep margins. Microscopically, the lesion demonstrated characteristic trichilemmal keratinization, marked nuclear atypia, focal tumor necrosis, and a high mitotic index of 14 per 10 high-power fields. Immunohistochemical profiling was performed to resolve the diagnostic ambiguity with classical SCC; the tumor cells showed complete negativity for monoclonal cytokeratins AE13 and AE14 (Dako, Glostrup, Denmark), definitively ruling out SCC and confirming the adnexal trichilemmal nature of the neoplasm.

Due to the tumor size and high-risk histological features (high mitotic index and necrosis), the patient was referred for adjuvant radiation therapy following multidisciplinary tumor board review. Simulation was performed via a dedicated CT scan utilizing a 5-point immobilization thermoplastic mask (Orfit Industries, Wijnegem, Belgium). The clinical target volume (CTV) encompassed the tumor bed with a 1.5-cm safety margin. The irradiation was delivered using Volumetric Modulated Arc Therapy (VMAT; Elekta AB, Stockholm, Sweden). A hypofractionated radiotherapy protocol was prescribed, delivering a total dose of 45 Gy in 10 fractions (4.5 Gy per fraction). Treatment was administered between January 29, 2026, and February 11, 2026. The therapy was well tolerated with no acute high-grade toxicity, and a satisfactory therapeutic outcome was achieved with no clinical or radiological recurrence at six months of follow-up (Figure 4).

**Figure 4:**
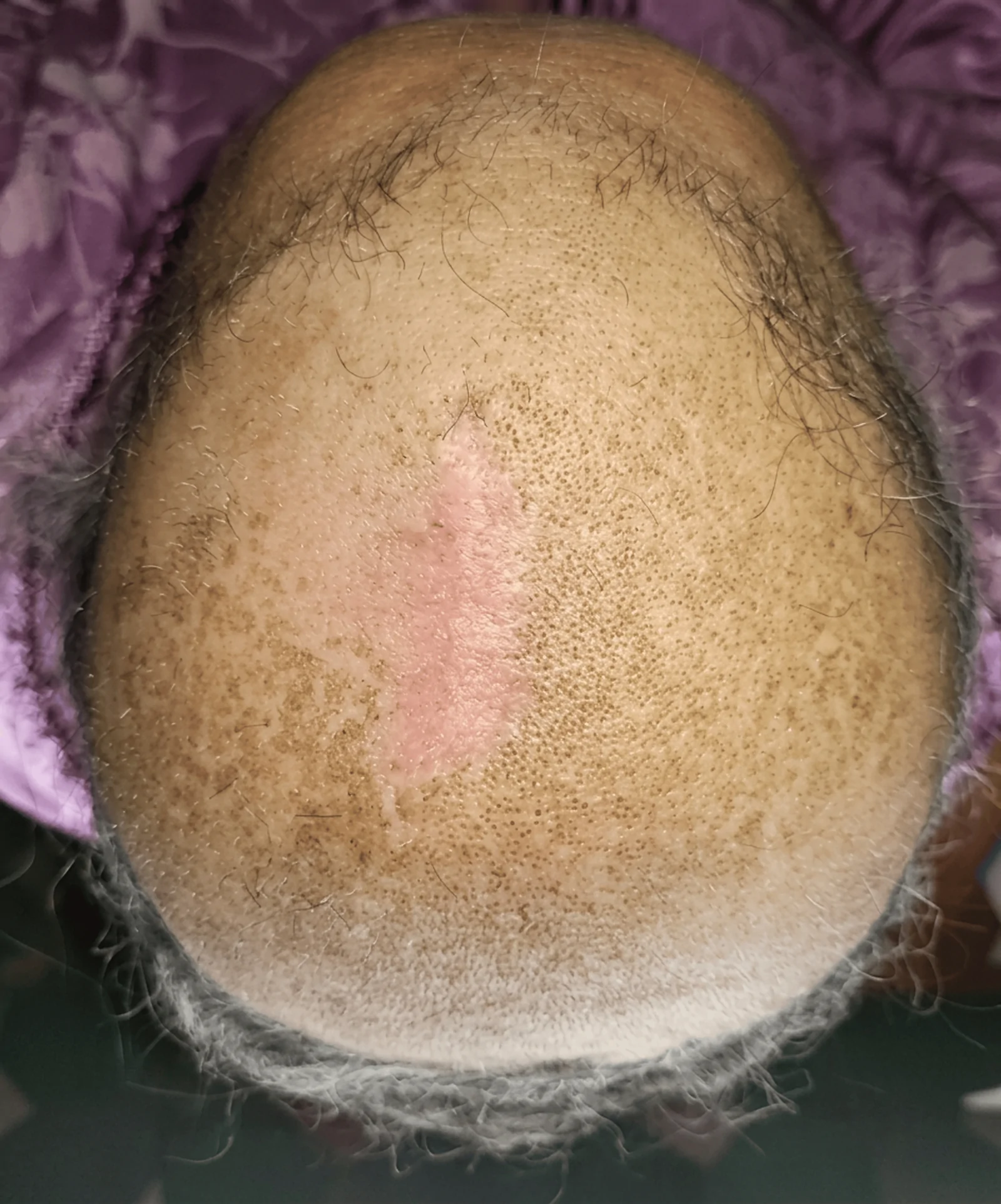
Clinical follow-up at six months post-radiotherapy.

## Discussion

MPTT is a rare adnexal malignancy, initially described by Wilson Jones in 1966. It predominantly affects the scalp, forehead, and cervical region, with a predilection for women over 40 years of age [5]. Clinically, MPTTs present as polypoid, ulcerated, or exophytic tumors [6]. This was observed in our patient, who presented with a painless, exophytic, and pedunculated scalp tumor that bled upon contact. These tumors can reach a considerable volume and invade surrounding structures, particularly the cranial bones, with a risk of cerebral involvement. In this case, the initial clinical examination showed no signs of locoregional or distant invasion, and no lymphadenopathy was detected.

A craniofacial CT scan can be essential for diagnosis, especially if cranial or cerebral involvement is suspected or if neurological signs are present. In our case, a CT scan was performed to confirm the absence of cranial involvement; it revealed a spontaneously hyperdense, heterogeneous mass measuring 75 mm along its major axis, showing heterogeneous contrast enhancement without bone lysis. Ultrasonography may also be requested to screen for lymphadenopathy. Additionally, MRI can show poorly marginated, ovoid, soft-tissue masses that are isointense on T1-weighted imaging and hyperintense on T2-weighted imaging relative to muscle [7].

The histological differential diagnosis often involves SCC. In our case, the preoperative biopsy was initially suggestive of SCC. However, the presence of trichilemmal keratinization, marked nuclear atypia, high mitotic rate, and the absence of AE13 and AE14 monoclonal antibody expression (Dako) allowed for the definitive exclusion of SCC [6].

The treatment of choice for MPTT is wide surgical excision with a 1-cm margin and deep periosteal margin verification [8]. Our patient underwent wide excision under general anesthesia. Regarding the closure of the large resulting defect, primary anatomical repair was successfully guided by extensive subgaleal undermining and serial galeal scoring to relieve mechanical tension, followed by a meticulous two-layer suture. An intraoperative drain was placed and removed the following day, leading to a satisfactory clinical outcome.

Following a multidisciplinary tumor board decision, the patient was referred for adjuvant radiation therapy due to high-risk features (tumor size > 5 cm, high mitotic rate, and aggressive histological nature) despite clear surgical margins. In our case, a hypofractionated VMAT protocol of 45 Gy in 10 fractions was successfully completed with excellent tolerance. Hypofractionated radiation schedules are well-established in high-risk cutaneous malignancies, providing high biologically effective doses to mitigate locoregional recurrence risks while minimizing toxicity to underlying intracranial structures and reducing overall treatment duration [9]. In cases of MPTT, adjuvant radiotherapy has shown favorable local control when combined with wide surgical resection, particularly for large or high-grade lesions.

A primary limitation of this report is the six-month follow-up period. Given the potential for late locoregional recurrence or distant metastasis reported in retrospective literature and systematic reviews, rigorous long-term clinical and radiological surveillance remains mandatory.

## Conclusions

MPTT remains an exceptionally rare cutaneous adnexal malignancy that clinical teams must thoroughly understand to avoid misdiagnosis, particularly with SCC. Accurate preoperative evaluation and extensive definitive histopathological examination using immunohistochemistry remain the cornerstones of effective management. Wide surgical excision with negative margins remains the primary treatment. In high-risk cases, tailored adjuvant hypofractionated radiotherapy was well-tolerated and associated with no clinical recurrence during the six-month follow-up period. While evidence supporting postoperative radiotherapy in MPTT is currently limited to retrospective case series and reports, adjuvant therapy may be considered for aggressive lesions, though longer follow-up is required to confirm long-term oncological control.
